# Companion Animals, Natural Disasters and the Law: An Australian Perspective

**DOI:** 10.3390/ani2030380

**Published:** 2012-08-27

**Authors:** Steven White

**Affiliations:** Griffith Law School, Griffith University, Brisbane, Queensland, 4111, Australia; E-Mail: steven.white@griffith.edu.au

**Keywords:** companion animals, pets, natural disasters, law, property, Australia

## Abstract

**Simple Summary:**

One of the issues raised by recent natural disasters in Australia is the management of companion animal welfare in disaster planning, response and recovery. Official inquiries following these disasters uncovered a number of shortcomings in addressing the management of animal welfare issues. This article suggests that despite some reform following these events, disaster management still fails to take seriously the interests of companion animals.

**Abstract:**

This article examines the regulation of companion animal welfare during disasters, with some context provided by two recent major disaster events in Australia. Important general lessons for improved disaster management were identified in subsequent inquiries. However, the interests of companion animals continue to be inadequately addressed. This is because key assumptions underpinning disaster planning for companion animals—the primacy of human interests over animal interests and that individuals will properly address companion animal needs during times of disaster—are open to question. In particular these assumptions fail to recognise the inherent value of companion animals, underestimate the strong bond shared by some owners and their animals and, at the same time, overestimate the capacity of some owners to adequately meet the needs of their animals.

## 1. Introduction

Australia has experienced a number of significant natural disasters over the past few years, most notably bushfires in Victoria in 2009, in which 173 people lost their lives, and major flooding in Queensland in 2010–11, claiming the lives of 33 people. Given the scale of these events, and the risk that such events could become more frequent due to the effects of climate change, significant time and effort has been invested in exploring the origins of these individual events, the effectiveness of the respective emergency responses, and ways in which the risks of these types of event, and responses to them, could be better managed in the future. It is notable, though, that in the inquiries addressing these events, and the recommendations for future change emanating from them, there is relatively little consideration of the issues raised by the presence of companion animals in the lives of those at risk of being affected by natural disasters. This might suggest that there has already been thorough consideration of the management of companion animals during and in the aftermath of natural disasters. However, submissions by animal welfare agencies and organisations such as the Australian Veterinary Association suggested otherwise. As this article will show, the Victorian bushfires disaster highlighted significant shortcomings in animal welfare management. The State Government has sought to address these shortcomings through the development and implementation of a Victorian Emergency Animal Welfare Plan, the most comprehensive emergency planning document addressing animal welfare in the country. By contrast, despite similar coordination issues emerging in the Queensland floods disaster, there has been a much less comprehensive response to animal welfare management. Regardless of these different responses, in both jurisdictions the emphasis is placed on prioritising human life over animal life, implicitly assuming there is a possible conflict between the two. As well, the onus is squarely placed on individuals to take responsibility for dealing with their companion animals during and in the aftermath of a disaster event.

This article will argue that the assumptions underpinning the management of companion animals in disaster situations can be criticised on at least three grounds. First, they fail to recognise the intrinsic value of companion animals as beings worthy of care and respect in their own right. Second, they overlook the extent to which companion animals are now considered by a high proportion of Australian households to be ‘members of the family’, and the expectations this might create about disaster planning arrangements which address the needs of all ‘family members’, human and companion animal alike. Third, even in day to day life it is clear that not all individuals are capable of adequately caring for their companion animals, so the expectation that all individuals will properly address the needs of their companion animals during a high-stress event such as a disaster is clearly not realistic. 

The legal system is implicated in the regulatory failure to adequately address the interests of companion animals in disaster situations, since there is a paucity of law which addresses their management in these situations. This situation arguably reflects, to a greater or lesser extent, the underlying property status of all domesticated animals in law. The strict legal categorisation of companion animals as personal property, or things, rather than legal persons, infuses much natural disaster planning in Australia.

While this article is focussed on the management of companion animals during disasters, this is not to deny, and is not inconsistent with, the importance of ensuring that the needs of humans are addressed as well as they can be in such situations. Meeting the needs of humans and companion animals in natural disaster situations is not incompatible and, given the status accorded to many companion animals of ‘family member’, may well be necessary to achieve the best possible outcome for both. 

As a final preliminary matter, a focus on companion animals does not imply that the needs of other animals, especially farm animals and wild animals, are not equally important. A key reason for focussing on companion animals is their emotional significance for humans. They enjoy the strongest level of animal welfare protection of all categories of animal, at least in formal legal terms. If there are valid concerns which can be raised about how well the needs of companion animals are being addressed in disaster management, these concerns will be magnified for other animals, especially farm animals.

## 2. Recent Major Disasters in Australia and Institutional Responses

Two recent major disasters in Australia saw the loss of human and non-human animal life, and major property destruction.

### 2.1. 2009 Victorian Bushfires

In the state of Victoria the so-called ‘Black Saturday’ bushfires erupted on 7 February 2009. In the last week of January 2009 Victoria had experienced one of the longest and hottest heatwaves on record. High temperatures continued into February. Conditions on 7 February 2009 were marked by record or near-record temperatures, and shifting, strong winds. Some fires had commenced prior to 7 February, others on the day. The Final Report of the 2009 Victorian Bushfires Royal Commission summarised the disaster as follows:

*The bushfires of late January and February 2009 had a devastating impact on Victoria. Apart from the loss of life, public hospitals provided emergency care to more than 800 people and admitted more than 130 people with a fire-related injury or illness. The fires also destroyed or damaged privately and publicly owned property and infrastructure, national parks, livestock and wild animals*.

[1] (p. 342)

The loss of life was significant:

*Five of the 13 major fires that burned in early 2009 led to loss of human life—Beechworth-Mudgegonga, Bendigo, Churchill, Murrindindi and Kilmore East. The greatest loss of life resulted from the Kilmore East fire (119 people), followed by Murrindindi (40), Churchill (11), Beechworth-Mudgegonga (2) and Bendigo (1). Nearly all these people died on 7 February itself; four died in the succeeding days or weeks as a result of the injuries they sustained on 7 February, and one person died as a result of injuries sustained after 7 February*.

[1] (p. 235)

The Bushfires Royal Commission was established by the State Government, with wide terms of reference, publishing interim reports in August 2009 and November 2009 and a final report in July 2010. 

The Victorian Royal Commission addresses the presence of animals in the disaster, acknowledging ‘the losses—of family, friends, fellow citizens, homes, gardens, animals, and the many other things that people hold dear’ [2] (p. vii). The Report also notes that the major animal welfare organisation in Victoria, RSPCA Victoria, estimated that more than 1 million animals were killed [1] (p. 343). Although not specified, these are likely overwhelmingly to be native wild animals, and to a lesser extent farm animals (the Department of Primary Industries estimated that 8,156 farm animals were killed by the fires or killed by vets and other staff due to burn injuries) [3] (p. 2). RSPCA Victoria, in its submission to the Royal Commission, made some important observations, including that ‘[a]nimal life and human life should not be considered mutually exclusive’. The RSPCA reported that ‘[i]t became clear to RSPCA staff working in the fire affected areas that many people placed the safety of their animals above their own wellbeing . . . The bush fire experience reconfirmed to RSPCA staff the extreme care and affection many people have for their animals—the grief when an animal is lost and the joy when an animal was found’ [4], (pp. 4,5).

Both the RSPCA and Animal Aid, a local animal welfare organisation, identified significant problems in coordinating services for animals, inadequate consideration of companion animals in emergency planning, and problems in care and identification in the days and weeks following the bushfires. Animal Aid reported that:

*Some relief centres were established in buildings and were unable to effectively house evacuated pets resulting in people being turned away or having to abandon their animals. For people who regard their animals as family members, this was not acceptable . . . In some cases people delayed leaving their homes or placed their own lives in danger to rescue their pets from the fire*.

[5] (p. 12)

In a detailed submission to the Royal Commission, the Australian Veterinary Association (AVA) also identified a lack of preparation and the lack of a coordinated response in addressing the interests of animals:

*The most concerning observation was that there was extremely poor planning, coordination and communication in relation to the response for animals affected by the fires. All agencies and organisations involved, including the AVA but possibly with the exception of the Victorian Department of Primary Industry, were clearly overwhelmed by the magnitude of the event and as a consequence of grossly inadequate planning for disaster management there was a lack of coordination of the response, particularly in relation to pets, horses and wildlife*.

[6] (p. 15)

The AVA also noted that many companion animals perished during the fires:

*The extent of veterinary attention required for pets and horses that had escaped the fires was also unprecedented. In relation to pets, this was to be expected given the extent of property loss, and the many thousands of people that had escaped the fires and were left homeless. Fortunately most of these people escaped with their pets. However many pets were burned and injured or perished in the fires*.

[6] (p. 2)

Consistent with the reports of animal welfare agencies and the AVA, the Victorian Department of Primary Industries (DPI), the main government organisation responsible for domesticated animal welfare matters, acknowledged major shortcomings in leadership and coordination. There was confusion about which agencies or organisations should be responsible for managing animal welfare matters. The DPI acknowledged that:

*Many organisations looked to DPI for the leadership and direction needed to enable coordination of efforts to address animal welfare needs caused by the February 2009 fires. The need for a leadership and coordination role for animal welfare operations across all species has not been apparent in previous emergencies . . .*.

[3] (p. 16)

The Final Report of the Royal Commission acknowledges the significance of companion animals to their owners, and echoed a recommendation from the DPI that greater clarity be provided for agency responsibility for animal welfare during emergencies:

*There does not appear to be a coordinated approach to animal welfare during relief operations. Improving agency coordination would help to provide more effective relief to all animals regardless of whether they are wildlife, stock, companion animals or pets. There is a good argument to address the welfare of all animals holistically in the *Emergency Management Manual Victoria.

[7] (p. 345)

Finally, in addressing the shared responsibility for preparing for and responding to bushfires, the Royal Commission only specifically addresses companion animals in the context of *individual* responsibility for ‘deciding what to do with pets and other animals’ [7] (p. 353).

The State Government responded to the coordination problems identified in the Royal Commission Final Report by requiring the DPI to lead the preparation of a stand-alone animal welfare emergency management plan. The *Victorian Emergency Animal Welfare Plan* provides for a State Emergency Animal Welfare Unit, drawing in State government agency representatives, as well as representatives from the AVA and RSPCA [8]. The Plan sets out animal welfare services during an emergency, roles and responsibilities, and operating principles. The Plan also sets out ‘Guiding Principles’, two of which are that ‘[t]he safety and welfare of all people is the overarching priority at all times’ and ‘[t]he responsibility for the welfare of animals at all times remains with the person in charge of an animal’ [8]. With respect to the second of these principles, the Plan separately suggests that ‘[i]n the event of an emergency, Government acknowledges the supporting role it can play in helping owners or carers meet their requirements’ [8].

### 2.2. 2010-2011 Queensland Floods

In late 2010 and into early 2011 the state of Queensland was subject to serious flooding, resulting in the loss of life and significant property damage. The Commission of Inquiry established following the floods described the disaster in the following terms:

*Prolonged and extensive rainfall over large areas of Queensland, coupled with already saturated catchments, led to flooding of historic proportions in Queensland in December 2010, stretching into January 2011. Thirty-three people died in the 2010/2011 floods; three remain missing. More than 78 per cent of the state (an area bigger than France and Germany combined) was declared a disaster zone; over 2.5 million people were affected. Some 29 000 homes and businesses suffered some form of inundation. The Queensland Reconstruction Authority has estimated that the cost of flooding events will be in excess of $5 billion*.

[9]

The Commission of Inquiry published an interim report on 1 August 2011 and a final report on 16 March 2012. RSPCA Queensland, in a submission to the Inquiry, identified significant problems with coordination and resourcing in relation to companion animals and wild animals, and widely varying degrees of preparation at local government level [10] (p. 1). In particular, the RSPCA highlighted the total lack of consideration of animals in prior emergency planning:

*Although RSPCA was represented no real planning occurred with respect to animals. Issues that were not considered included: [w]here pet animals would go (no animal evacuation centre(s) identified); [h]ow they would be housed (no portable cages, food, food bowls ready to be deployed); [h]ow to deal with people who would not leave flooded or threatened areas without their pets (rescue people on the ground did not know what to so in these situations); [w]ho would pay for RSPCA rescue and emergency work*.

[10] (p. 2)

The Commission provided only cursory attention to the management of companion animals. The Interim Report noted that:

*During the 2010/2011 floods, some pet owners were reluctant to evacuate if they could not take or make arrangements for the care of their pets. This was made easier where councils had plans for sheltering pets, as for instance in Rockhampton, where the council worked with the RSPCA to shelter pets in a facility alongside the evacuation centre. Similarly the Ipswich City Council had an animal management team who were able to care for pets at the Ipswich showgrounds evacuation centre and the Lockyer Valley Regional Council worked closely with the University of Queensland Veterinary School at Gatton to care for domestic and farm animals*.

[11] (p. 197)

The Commission made recommendations that essentially reflected existing policy: ‘Councils, as part of their community education program for disaster preparation, should encourage pet owners to consider what they will do with their pets if they need to evacuate’; ‘Councils should work with the RSPCA to develop plans about transporting and sheltering pets should they need to be evacuated with their owners’; and ‘Animal shelters, zoos, stables, and similar facilities should develop plans for evacuating or arranging for the care of animals in consultation with their local council. Local disaster co-ordinators should be aware of what plans exist’ [11] (p. 198).

The Queensland Government, in responding to these recommendations, declined the opportunity to modify existing policy or approaches, stating that the recommendations required no action at a state level given they addressed matters for local government [12] (p. 41). This lack of State leadership, by contrast with that which has occurred in Victoria since 2009, suggests that Queensland will be much less well-prepared to address the interests of companion animals than it might otherwise be, given the particular problems highlighted by the RSPCA, as well as the coordination and recovery problems likely to occur in a disaster situation in which there may be less time to respond than in a flood.

## 3. Legal Issues Raised by Treatment of Companion Animals in Disasters

Having established the range of management issues which may arise with respect to the interests of companion animals, using two recent Australian disasters and subsequent government responses, this section of the article explores the specifically legal regulation of these issues. The management of companion animals during disasters is addressed in three broad areas of the law—liability law, animal welfare law and emergency management law. 

### 3.1. Liability Issues

There are a wide range of potentially significant legal issues raised by the treatment of companion animals in disaster situations. For example, Anderson and Anderson identified a number of legal questions which were researched in the United States by the Animal Legal Defense Fund (ALDF), in the aftermath of Hurricane Katrina [13]. These issues included:

- the position of rescuers who trespass or break into and enter people’s private property to rescue abandoned animals.
- whether veterinarians can treat, including sterilize, animals without owner consent following a disaster.
- where more than one person claims ownership to an animal recovered during a disaster, how those competing claims can be resolved.
- the role of the police and the military in rescuing or shooting abandoned animals.
- the position of animal welfare shelters in dealing with abandoned animals.

Drawing on a wide range of volunteer attorneys, the ALDF published a list of short answers to these questions, with fuller legal opinions supporting the short answers available on request [14]. No legal work of a similar nature has been undertaken in Australia. While the issues addressed by the ALDF are important, they are concerned primarily with liability issues for those dealing with animals during disasters, and not the well-being of animals *per se*. Of course, the answers to questions of potential liability will have indirect implications for animal welfare, to the extent they affect the way particular persons deal with animals during a disaster.

### 3.2. Animal Welfare Law

In Australia the States and Territories have primary legislative responsibility for addressing animal welfare. Local government also plays an important role, with its ambit increasingly extending beyond animal management issues to welfare issues as well. The Commonwealth plays a very limited legislative role in companion animal issues, although it is important in broader policy terms, especially through implementation of the *Australian Animal Welfare Strategy* (AAWS) [15]. The AAWS sets out future policy directions for animal welfare, including the need for national harmonisation of animal welfare law. The Commonwealth has acknowledged the importance of consideration of animal welfare in emergency preparedness, suggesting that ‘[i]t is important to recognise broad stakeholder and community animal welfare interests and the need to communicate agreed emergency response policies and approaches as part of emergency planning and preparedness’ [16]. However, the focus is limited to disease management and the revised AAWS does not address emergency management at all.

There are three aspects of State and Territory animal welfare legislation that may have some bearing on management of companion animals during disasters—cruelty offences, abandonment offences and duty of care requirements [17].

All State and Territory statutes include a prohibition against cruelty to an animal, with breach of the provision an offence punishable by a fine and/or imprisonment. Importantly, the cruelty prohibition is qualified in most jurisdictions. Generally, an act or omission is cruel so long as it is unjustifiable, unnecessary or unreasonable. For example, the Western Australian Act defines cruelty as causing unnecessary harm (*Animal Welfare Act 2002*, s 19(2)). In Queensland, cruelty includes causing pain that in the circumstances is unjustifiable, unnecessary or unreasonable (*Animal Care and Protection Act 2001*, s 18(2)(a)). In South Australia ‘ill-treatment’ includes unreasonably causing an animal unnecessary harm (*Animal Welfare Act 1985*, s 13(3)). Although not judicially tested, the scope of this qualification will be very important in circumstances where animals have been harmed by a human during a natural disaster, but for operational or other allegedly legitimate management purposes.

In some jurisdictions abandonment of an animal is generally prohibited as part of the cruelty prohibition, and so is subject to the same qualifications as cruelty generally. Other jurisdictions have a separate abandonment offence. This is usually qualified, as in Queensland, by reference to whether there is a reasonable excuse or authorisation by law for the abandonment (*Animal Care and Protection Act 2001*, s 19). Again, although not judicially tested, it’s clear that coping with the demands of a disaster will be relevant to the scope of a reasonable excuse.

Finally, duty of care provisions could potentially be relevant. Two jurisdictions—Queensland and Tasmania—impose an explicit duty of care on persons in charge of an animal (*Animal Care and Protection Act 2001* (Qld), s 17; *Animal Welfare Act 1993* (Tas), s 6). This entails a requirement to meet the basic welfare needs of an animal. Other jurisdictions have provisions which are similar in effect, even if they are not explicitly labelled a ‘duty of care’. All jurisdictions adopt similar qualifications, only requiring care that is appropriate or reasonable in the circumstances. Again, these sorts of qualifications will be particularly relevant in a disaster situation. The legislation in Queensland explicitly bears this out. After setting out the content of the duty of care in s 17(3) of the *Animal Care and Protection Act 2001* (Qld), such as providing appropriate food and water, sub-section 17(4)(b) states that in deciding appropriateness regard has to be paid to the steps a reasonable person in the circumstances of the person would reasonably be expected to have taken. The draftsperson then cites, as an example of a relevant circumstance, a bushfire or another natural disaster, or a flood or another climatic condition.

In summary, animal welfare legislation may have limited application in circumstances of natural disaster. To the extent that it might be relevant, for example in providing a reasonable excuse for failing to meet the welfare needs of an animal, there is as yet no judicial consideration of the scope of such excuses. Perhaps most importantly, given its focus on the treatment of individual animals, animal welfare legislation does not address broader issues of collective planning for and management of animal welfare in disaster situations. 

### 3.3. Animal Welfare and Disaster Management Legislation

It might be expected that the management of animal welfare in disasters would be addressed in state and territory disaster management legislation. However, when disaster management legislation does address animals, and in many jurisdictions there is no reference to animals at all, it is usually in the context of the powers of authorised officers to deal with them. For example, under s 77 of the Queensland *Disaster Management Act 2003* a disaster coordinator can control the movement of persons, vehicles and animals in declared areas; they can evacuate persons or animals from declared areas; and they can contain an animal within an area, or remove and destroy an animal. Similar powers are found in some other jurisdictions, such as those in s 25 of the *Emergency Management Act 2004* in South Australia. New South Wales provides an exception. Section 37A of the *State Emergency and Rescue Management Act 1989* (NSW) provides for the Minister, in the circumstances of an emergency, to authorise an emergency services officer to take measures to protect an animal from injury or death. Even here, though, the circumstances are confined to making particular areas secure, rather than providing for ongoing management of the care of an animal.

Although the account provided here is a brief one, disaster management legislation does not address the welfare of animals in any meaningful way. This suggests a need to look beyond statutory regulation to regulation in a broader sense, especially state and local government disaster plans, and administrative arrangements entered into between government and non-government agencies. Few state disaster management guidelines or plans in the Australian states and territories address the welfare of animals in any detailed way. The major exception to this general principle is Victoria. As discussed above, the 2009 bushfires prompted the State Government to put in place a comprehensive emergency plan for animal welfare.

In summary, neither animal welfare law nor emergency management law address the management of the welfare of companion animals in disaster situations in any comprehensive way. Administrative regulation is also very limited, with the exception of Victoria’s recently implemented animal welfare emergency management plan. To the extent that the needs of animals are addressed they are subject to the overriding priority of human interests. And, as suggested in Part 2 of this article, the mantra of individual responsibility for the welfare of animals is a central principle in disaster regulation. These two features of disaster regulation are vulnerable to criticism on three grounds: they overlook the intrinsic value of companion animals; they underestimate the significance of companion animals in the lives of Australian families; and they overestimate the capacity of some individuals to competently meet the needs of their companion animals.

## 4. Valuing Companion Animals in Times of Disaster

### 4.1. The Intrinsic Worth of Companion Animals

It is notable that in addressing the welfare of companion animals in disasters none of the Royal Commission into the Victorian Bushfires, the Queensland Floods Commission of Inquiry, or subsequent institutional responses affirm the need to act on the basis that companion animals are intrinsically valuable as sentient beings. Such an affirmation is also absent from animal welfare law and disaster management legislation. As shown in Part 2, there is some recognition, especially in submissions to the respective inquiries, that companion animals may hold special significance for their owners. However, this is then framed as an issue of human management, to the extent that, for example, attachment to animals may affect the willingness of owners to leave their property or to enter evacuation centres not equipped to house animals. Such an approach overlooks the sentience of animals, and the ethical obligations this imposes on those whose responsibility is to care for them. Whether couched in terms of animal rights [18], animal interests [19], animal capabilities [20] or an ethic of care [21], the sentiency of companion animals provides the basis for arguing that policy and legal responses need to directly address the claims of companion animals to proper care in their own right, and not just as an incidental aspect of addressing human interests.

### 4.2. Companion Animals as Family Members

Companion animals are increasingly regarded by their owners as members of the family. Sociological research and opinion surveys show that a very high proportion of Australian households include at least one companion animal, and that an overwhelming majority of those households regard their companion animal as a member of the family [22]. Other Western jurisdictions, including the United States, show similar tendencies:

*In another Australian survey conducted in 1994 and repeated in 2006 by the Melbourne Institute of Applied Economic and Social Research, the National People and Pets Survey confirmed that 92 per cent of pet owners felt ‘very close’ to their pet. The bond between pet owner and companion animal is so great that some surveys in the United States have even revealed that 50 per cent of pet owners would be ‘very likely’ to risk their lives to save their pets, and another 33 per cent would be ‘somewhat likely’ to put their own lives in danger for the sake of their pets*.

[23] (p. 207)

In Australia, Franklin has confirmed that the high proportion of households describing their companion animals as ‘family members’ is not simply a matter of empty sentimentalism, given the access to all parts of the household routinely enjoyed by companion animals:

*The symbolism of household space needs to be emphasised here. Bedrooms are largely highly private spaces, the inner sanctum of privatised societies … in this sense when people in our survey stated that an animal was both a member of the family and allowed into their bedroom, it was a refined answer indicating that they were not just a member of the family but a very close intimate member … in the past when dogs were kept outside, or when they were allowed inside but not on furniture, their separate, inferior status was being marked. To discover that half of those interviewed allowed their animals on furniture is to uncover a major shift in their status and position relative to humans and human society*.

[24] (pp. 211,212)

Although, as will be suggested below, there is a duality at the heart of our relationship with companion animals such that claims to family status should not be accepted uncritically, it is clear that for many households companion animals enjoy recognition as family members. This is reflected not just in opinion surveys and sociological research surveys, but in the observed responses of humans in natural disaster situations, as reported, for example, in submissions to the Royal Commission into the Victorian Bushfires considered above. This has very important implications for policy and legal responses to the management of companion animals in disaster contexts. This depth of feeling for companion animals means that where adequate arrangements are not in place to address the needs of companion animals in traumatic situations such as a disaster, owners are likely to be further stressed and agitated, and prone to act in ways which may bring harm to themselves or others. It strongly supports the contention that addressing the needs of humans and companion animals equally should be a part of disaster planning, response and recovery, since it accords with the professed and actual preferences of a significant proportion of households and is consistent with recognition of the intrinsic value of humans and companion animals.

### 4.3. Discarding or Harming ‘Family Members’

As has been established in Part 2, the principle that individual owners are primarily responsible for the care of their animals in time of disaster is ubiquitous in emergency planning approaches. While this is usually supported by sensible suggestions about the need for adequate emergency preparation for the care of companion animals, and practical measures to achieve this, the principle assumes a great deal about the competency and commitment of some companion animal owners. It overlooks the dual nature of our relationship with companion animals. Many households regard their companion animals as family members. Perhaps consistent with this status, in Australia the formal legal protection for companion animals against cruelty and duty of care breaches is the highest of any category of animal [17]. On the other hand, significant numbers of companion animals are subject to cruelty and duty of care breaches every year, investigation and prosecution have been hampered by lack of resources and expertise, and sentencing courts have failed to take cruelty and duty of care breaches seriously [17]. Importantly, a close examination of the circumstances in which companion animals finish up in shelters shows that many are relinquished by owners for relatively trivial reasons:

*a significant number of companion animals are freely surrendered to animal shelters each year in Australia, largely for ‘owner-centric’ reasons. The fate of many of these animals—including young, healthy animals—is death. These companion animals are legally discarded, with no regulatory sanction falling upon those who relinquish their animals. There is, therefore, a striking tension in the way society regards companion animals. On the one hand, they are affectionately regarded as members of the family. On the other hand, the role of animal shelters shows that they are also regarded as dispensable, being freely discarded in significant numbers each year*.

[22] (p. 869)

The common-place nature of relinquishment and the significant number of welfare breaches reported each year in Australia suggest that in times of great stress, such as that of a natural disaster, reliance on individual owners to responsibly address the needs of their companion animals may be misplaced. Given the available empirical evidence about the ways in which some companion animals are routinely treated by their owners, it is naïve at best to rely on appeals to individual responsibility as a key component of disaster planning for companion animals. Much more is required:

*incorporating animals into disaster response is a positive step, but more basic steps in educating people about responsible guardianship might go further to reduce the hazards that animals face in future disasters. “Responsible” guardianship must go beyond simply providing food, water, and shelter. It must involve acknowledging a lifelong commitment, and fighting against threats to that commitment*.

[25]

Implicit in this argument, and in earlier arguments in this article, is the recognition that companion animals are deserving of respect as sentient beings, capable of suffering and also of flourishing, and that the obligation to meet their needs is a serious and long-lasting one. This, of course, is inconsistent with the underlying premise of the law, which classifies animals as the ‘personal property’ of their owners [26]. In formal legal terms, and in practice, animals are chattels, to be bought and relinquished at the whim of their human owners. While the intervention of animal welfare statutes shows that these property rights are not unqualified, an understanding of the property status of animals can help to illuminate why it is that the interests of companion animals are not addressed in disaster situations in a way consistent with their ethical claim to inherent value [27]. 

## 5. Conclusions

This article has sought to focus on the regulation of companion animal welfare during disasters, with some context provided by two recent major disaster events in Australia. Important lessons for improved general emergency management were identified in subsequent inquiries. However, the interests of companion animals continue to be inadequately addressed. This is because key assumptions underpinning disaster planning for companion animals—the primacy of human interests over animal interests and the ability of individuals to properly address companion animal needs during times of disaster—are open to question. In particular they fail to recognise the inherent value of companion animals, underestimate the strong bond shared by some owners and their animals and, at the same time, overestimate the capacity of some owners to adequately meet the needs of their animals. And, if companion animals arguably enjoy the highest level of legal protection of all animals, the implications for less well-protected animals during times of disaster, especially farm animals, are even more stark.

The management of animals in disaster situations, including its expression through regulation, is an under-researched area in Australia. Much more research is required to more fully understand the range of settings in which animals are vulnerable to disasters, and the measures which might be taken to address these. This might contribute to a more serious engagement with these issues by institutions armed with the power to recommend relevant changes to regulation and those with the power to implement them. 

A number of questions, of relevance not only for lawyers but for all those concerned with the well-being of companion animals, immediately suggest themselves. First, is there a need for directive legislation specifically requiring government at all levels to address the issue of companion animal management in disasters, not as an after-thought in disaster management planning, but as a key component, and in a way which recognises the inherent value of companion animals and the responsibility we have for their well-being? Second, in a related question, is there a need for a broadly consistent approach, so that the welfare needs of animals are addressed in the same way, regardless of the particular jurisdiction they happen to be located in [28]. Third, it seems that the law provides little if any guidance on the respective roles of the various institutional actors preparing for and responding to the needs of companion animals in disaster situations. Should these roles be clarified in law, given the potential this brings for greater consistency, transparency and accountability? And what should their respective roles be? Finally, to move beyond a narrow disaster management setting, is there a need to rethink the prevailing legal status of companion animals, to shift away from their current classification as personal property, to some other legal status which more closely accords with their legitimate ethical claims on us?
